# Tetrafluoroborate‐Induced Reduction in Defect Density in Hybrid Perovskites through Halide Management

**DOI:** 10.1002/adma.202102462

**Published:** 2021-07-04

**Authors:** Satyawan Nagane, Stuart Macpherson, Michael A. Hope, Dominik J. Kubicki, Weiwei Li, Sachin Dev Verma, Jordi Ferrer Orri, Yu‐Hsien Chiang, Judith L. MacManus‐Driscoll, Clare P. Grey, Samuel D. Stranks

**Affiliations:** ^1^ Cavendish Laboratory Department of Physics University of Cambridge JJ Thomson Avenue Cambridge CB3 0HE UK; ^2^ Department of Chemistry University of Cambridge Lensfield Road Cambridge CB2 1EW UK; ^3^ Department of Materials Science & Metallurgy University of Cambridge 27 Charles Babbage Road Cambridge CB3 0FS UK; ^4^ Department of Chemical Engineering & Biotechnology Philippa Fawcett Drive Cambridge CB3 0AS UK; ^5^ Present address: Department of Chemistry Indian Institute of Science Education and Research Bhopal Bhopal Bypass Road, Bhauri Bhopal Madhya Pradesh 462066 India

**Keywords:** charge‐carrier recombination, defects, perovskite solar cells, photoluminescence, surface treatment, tetrafluoroborate

## Abstract

Hybrid‐perovskite‐based optoelectronic devices are demonstrating unprecedented growth in performance, and defect passivation approaches are highly promising routes to further improve properties. Here, the effect of the molecular ion BF_4_^−^, introduced via methylammonium tetrafluoroborate (MABF_4_) in a surface treatment for MAPbI_3_ perovskite, is reported. Optical spectroscopy characterization shows that the introduction of tetrafluoroborate leads to reduced non‐radiative charge‐carrier recombination with a reduction in first‐order recombination rate from 6.5 × 10^6^ to 2.5 × 10^5^ s^−1^ in BF_4_^−^‐treated samples, and a consequent increase in photoluminescence quantum yield by an order of magnitude (from 0.5 to 10.4%). ^19^F, ^11^B, and ^14^N solid‐state NMR is used to elucidate the atomic‐level mechanism of the BF_4_^−^ additive‐induced improvements, revealing that the BF_4_^−^ acts as a scavenger of excess MAI by forming MAI–MABF_4_ cocrystals. This shifts the equilibrium of iodide concentration in the perovskite phase, thereby reducing the concentration of interstitial iodide defects that act as deep traps and non‐radiative recombination centers. These collective results allow us to elucidate the microscopic mechanism of action of BF_4_^−^.

## Graphical abstract

The surface treatment of MAPbI_3_ through methylammonium tetrafluoroborate (MABF_4_) leads to reduced non‐radiative charge‐carrier recombination with a reduction in first‐order recombination rate by an order of magnitude. Solid‐state NMR is used to elucidate the atomic‐level mechanism of the BF_4_^−^‐additive‐induced improvements, revealing that it can act as a scavenger of excess methylammonium iodide (MAI).

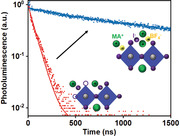

## 1 Introduction

The field of hybrid perovskite solar cells (PSCs) is one of the most rapidly growing in the history of photovoltaics. Hybrid PSCs have now achieved more than 25% photoconversion efficiency since the first report by Kojima et al. in 2009.^[^ ^1^, ^2^ ^]^ Hybrid perovskite materials have the potential to achieve world‐leading efficiencies in solar cell and light‐emitting diode devices due to their astonishing photophysical properties, such as long carrier diffusion lengths,^[^ ^3^ ^]^ high photoluminescence quantum yields (PLQY),^[^ ^4^ ^]^ bandgap tunability,^[^ ^5^ ^]^ and facile processability.^[^ ^6^, ^7^, ^8^, ^9^ ^]^ However, there remain obstacles to attaining optimal PSCs. In spite of some degree of defect tolerance, the prevalence of defects that lead to non‐radiative recombination still hinder the development of more efficient and more stable hybrid perovskite materials.^[^ ^10^ ^]^ Monitoring radiative recombination through luminescence techniques is an excellent probe of the impact of defects on optoelectronic behavior and a proxy for device performance, with any non‐radiative recombination leading to power losses in solar cells.^[^ ^11^ ^]^

There are a number of types of defects present in polycrystalline thin film perovskites used in PSCs, such as undercoordinated metal cations (Pb^2+^), interstitial halides (I^−^, Br^−^, etc.), organic cation (methylammonium (MA), formamidinium etc.) vacancies, and metallic lead (Pb^0^) clusters.^[^ ^12^ ^]^ Various routes based on coordinate,^[^ ^13^ ^]^ ionic,^[^ ^14^ ^]^ hydrogen,^[^ ^15^ ^]^ and halogen bonding^[^ ^16^ ^]^ have been proposed to overcome the aforementioned defects and associated disorder in hybrid perovskite materials, and thereby improve the performance of perovskite based opto‐electronic devices. An efficient way to passivate defects in hybrid perovskites is through ionic bonding with inorganic/organic halide salts such as KI,^[^ ^14^ ^]^ NaCl,^[^ ^17^, ^18^ ^]^ MAI,^[^ ^19^ ^]^ and phenylethyl ammonium iodide.^[^ ^15^ ^]^ Addition of these salts to the perovskite precursor solution, or by surface and grain boundary treatment, leads to enhancements in opto‐electronic properties and device performance.^[^ ^12^, ^20^ ^]^

The introduction of BF_4_^−^ has been shown recently to be beneficial for device performance and stability of halide perovskites. For example, improvement in the performance of solar cells and the long‐term stability of devices has been reported by using BF_4_^−^‐containing ionic liquids in the precursor solution such as 1‐butyl‐3‐methylimidazolium tetrafluoroborate ([BMIM]BF_4_).^[^ ^21^, ^22^ ^]^ We have previously shown that modification of MAPbI_3_ with BF_4_^−^ leads to changes in frequency‐dependent electrical conductivity and suggested that this may be due to incorporation of BF_4_^−^ into the 3D perovskite lattice.^[^ ^23^ ^]^ In the same vein, Yang et al. have recently shown that MAPbBr_3_ synthesized with BF_4_^−^ exhibits longer charge carrier lifetimes compared to the pristine perovskite.^[^ ^24^ ^]^ However, the atomic‐level mechanism of the modulation imparted by BF_4_^−^, including conclusive evidence for or against its incorporation in the perovskite structure, has been elusive and remains a topic of hot debate.^[^ ^21^, ^23^, ^25^, ^26^ ^]^ Solid state NMR (ssNMR) is an ideal tool to study the mechanism of BF_4_^−^ modulation as it is an element‐specific probe of local structure. ssNMR has previously been used to determine various phenomena in lead halide perovskites at the atomic level,^[^ ^27^, ^28^, ^29^ ^]^ including cation incorporation, phase segregation,^[^ ^30^, ^31^, ^32^ ^]^ and halide mixing.^[^ ^33^, ^34^ ^]^

In this work, we introduce BF_4_^−^ in the form of methylammonium tetrafluoroborate, CH_3_NH_3_BF_4_ (MABF_4_), as a molecular modulator for hybrid halide perovskites. The introduction of BF_4_^−^ leads to a considerable increase in the photoluminescence quantum efficiency (PLQE) and charge carrier lifetimes of MAPbI_3_, which is consistent with a reduction in trap density by over an order of magnitude. We elucidate speciation of BF_4_^−^ in the resulting compositions using multi‐nuclear (^19^F, ^11^B, ^14^N) magic‐angle‐spinning (MAS) ssNMR, which allows us to establish the microscopic mechanism leading to the reduction in trap density imparted by BF_4_^−^. We find that BF_4_^−^ is not incorporated into the perovskite structure, but instead acts as a scavenger of unreacted MAI. The removal of trace unreacted MAI, driven by the thermodynamic stability of a MAI–MABF_4_ cocrystal, decreases the concentration of interstitial iodide defects in the perovskite, which act as deep traps, and thus substantially decreases non‐radiative recombination. Consequently, we find that the BF_4_^−^ treatment reduces the deep trap density by a factor of ≈26 without significantly altering the intrinsic radiative properties of the perovskite. Such reductions in trap density may also explain the enhanced device stability.^[^ ^10^ ^]^

## 2 Results and Discussion

**Figure 1:**
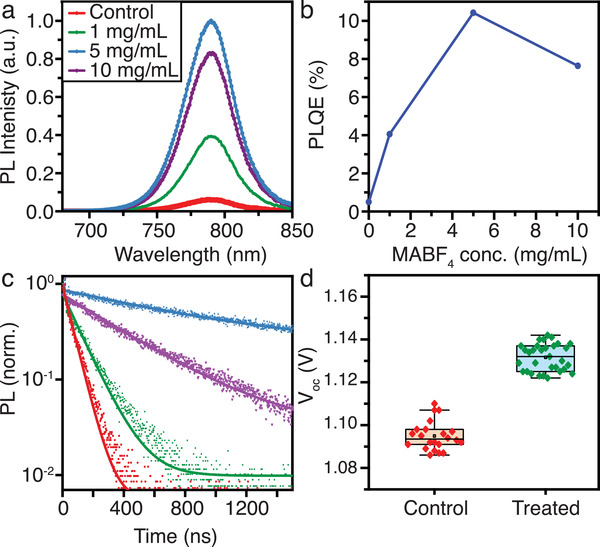
Optical Characterization. a) Normalized steady‐state PL spectra with excitation at 470 nm, b) PLQE with continuous wave excitation at 520 nm and an illumination intensity of 60 mW cm^−2^, and c) normalized time‐resolved PL decay curves (excited at 407 nm with a fluence of 5 nJ cm^−2^ per pulse and 0.5 MHz repetition rate) of MAPbI_3_ perovskites with and without post‐treatment with different concentrations of MABF_4_. The solid curves in (c) are fitted monoexponential decays with a constant background term (see Figure S5, Supporting Information, for the extracted time constants), which is justified at low excitation densities.^[^ ^35^ ^]^ d) Open‐circuit voltages (*V*_oc_) from Cs_0.05_(MA_0.17_FA_0.83_)_0.95_Pb(I_0.83_Br_0.17_)_3_‐based solar cells with no treatment (Control, 0 mg mL^−1^) and treated with MABF_4_ (Treated, 1 mg mL^−1^).

MAPbI_3_ perovskite films were prepared by spin‐coating precursor solutions on glass substrates, followed by annealing. Afterward, a post‐treatment was performed on selected samples by spin coating a solution of MABF_4_ in isopropyl alcohol (IPA) on the film surface (see Experimental Section). No significant changes to the perovskite structure were observed following treatment, and slight morphology changes and secondary phases only become evident at very high additive concentrations (see Figure S1, Supporting Information, for X‐ray diffraction (XRD) patterns and Figure S2, Supporting Information, for scanning electron microscopy images). We explored the strategy of adding MABF_4_ to the perovskite precursor solutions, but formation of undesired phases (visible white precipitation at higher concentration (>5%) of MABF_4_) encouraged us to pursue the post‐treatment route. In order to study the effect of MABF_4_ post‐treatment on the radiative recombination pathways in MAPbI_3_ films, PL spectroscopy measurements were performed. **Figure** **1**a shows photoluminescence (PL) spectra with 470‐nm excitation of a control MAPbI_3_ sample, along with films treated with different concentrations (1, 5, and 10 mg mL^−1^) of MABF_4_ in IPA. No significant spectral shift is observed, and the relative shape of the PL spectra remains unchanged (Figure S4, Supporting Information); together with negligible changes in the UV–Vis absorption spectra across the control and treated samples (Figure S3, Supporting Information), these observations indicate that there is no change in bandgap or film thickness with treatment. However, the PL intensity clearly increases upon BF_4_ treatment, reaching a maximum for a concentration of 5 mg mL^−1^ that is a factor of 16 larger than the integrated intensity of the control sample. These improvements are quantified in Figure 1b, which shows that the external PLQE of the films under 1‐sun‐equivalent illumination conditions increases from 0.5% for the control film to 10.4% for the best‐performing treated sample (5 mg mL^−1^). Complementary time‐resolved kinetic measurements of the emission at the perovskite PL maximum (≈790 nm) with the different BF_4_^−^ treated MAPbI_3_ films are shown in Figure 1c. These measurements were performed at low fluence (≈5 nJ cm^−2^ per pulse), corresponding to an initial excitation density of ≈3 × 10^14^ cm^−3^, to ensure recombination was in the trap‐limited, monomolecular regime.^[^ ^35^ ^]^ It can clearly be seen that upon treatment the charge‐carrier lifetime increases, with the monoexponential decay constant rising from 66 ns to a maximum of 952 ns for post‐treatment with 5 mg mL^−1^ BF_4_^−^, consistent with the PLQE trends (see Figure S5, Supporting Information, for the extracted lifetimes). The increased PLQE with post‐treatment, concomitant with increased lifetime in the low‐fluence regime, is associated with the reduction in trap‐mediated, non‐radiative charge‐carrier recombination.^[^ ^36^ ^]^ These luminescence enhancements also translate into open‐circuit voltage increases in full solar cells employing MAPbI_3_ with treatment (0.98 ± 0.01 V for a treatment concentration of 1 mg mL^−1^, optimized for devices) compared to the untreated controls (0.92 ± 0.02 V) (Figure S6; see Supporting Information for experimental details). Similar enhancements are also seen when treating triple‐cation [Cs_0.05_(MA_0.17_FA_0.83_)_0.95_Pb(I_0.83_Br_0.17_)_3_] absorber layers (*V*_oc_ = 1.13 V ± 0.01 V) compared to the control (1.09 V ± 0.01 V) (Figure 1d, Figure S7 and Table S1, Supporting Information).

### 2.1 Modeling PL Dynamics

**Figure 2:**
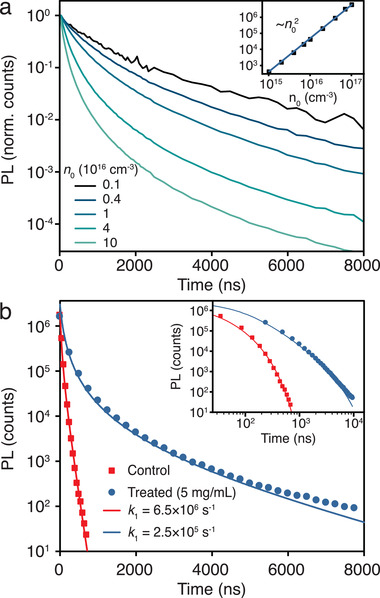
Transient PL dynamics and modeling. a) Excitation‐dependent PL kinetics of the 5 mg mL^−1^ post‐treated sample normalized by intensity at time‐zero. Initial carrier densities are calculated as outlined in the Supporting Information. Inset: Initial PL intensity versus injected carrier density at time‐zero, *n*_0_. The blue line is a fit showing that the initial PL intensity scales with $n_{0}^{2}$. b) Spectrally integrated PL decays of the control and 5 mg mL^−1^ post‐treated samples. Solid lines are modeled PL decay curves of the form shown in equation 2. The *k*_1_ parameter stated is significantly modified by the BF_4_^−^ post‐treatment (see Table S2, Supporting Information, for model parameters).

To further study the effect of the MABF_4_ additive on the overall carrier recombination dynamics, fluence‐dependent spectrally resolved time‐resolved photoluminescence (TRPL) measurements were performed on the MAPbI_3_ control sample and the most emissive treated sample (5 mg mL^−1^ MABF_4_) over a range of excitation densities. We note that no spectral shifts are observed during the photoluminescence decay of either sample (see Figure S8, Supporting Information, for the time‐resolved spectra). **Figure** **2**a shows the spectrally integrated PL kinetics of the treated sample normalized to the intensity at time zero, with initial excitation densities (*n*_0_) ranging from ≈10^15^ to 10^17^ cm^−3^. The initial PL decay rate increases with increasing injection rate across the whole fluence range, indicating that the decay kinetics in the carrier density regime *n* > 10^15^ cm^−3^ are dominated by higher order recombination processes beyond first order. The inset of Figure 2a shows that the initial PL intensity scales with $n_{0}^{2}$ across the fluence range, confirming that radiative recombination proceeds through bimolecular recombination of electrons and holes, as expected for an intrinsic semiconductor with no significant charge doping concentration compared to the photoexcited carrier density.^[^ ^37^ ^]^

To quantify the defect mitigation caused by the BF_4_^−^ treatment, and to give an overall picture of recombination, we modeled the recombination dynamics. As with any semiconductor, the carrier recombination processes in halide perovskites may be categorized by their first, second, and third order dependencies on the carrier density, *n*. The generic rate of change of the carrier population can be described as:

(1) $$\;\frac{dn}{dt}=G-k_{1}n-k_{2}n^{2}-k_{3}n^{3}$$

where *G* is the generation rate and *k_i_* is the *i* ^th^‐order rate constant.^[^ ^38^ ^]^ We neglect the third‐order Auger (*k*_3_) term^[^ ^39^ ^]^ as this component is only relevant in MAPbI_3_ at excited carrier densities above 10^17^ cm^−3^.^[^ ^40^, ^41^ ^]^ We also exclude the contribution of lateral carrier diffusion since the profile of the laser excitation precludes this (see Experimental Section), and assume that the diffusion throughout the thickness of the 300‐ nm film is rapid on the timescale of the measurements.^[^ ^42^ ^]^ On the basis that the samples are intrinsic in nature, the intensity of the PL signal as a function of time goes as:

(2) $$PL\left(t\right)\approx {\left(\frac{n_{0}k_{1}}{n_{0}{k^{\prime }}_{2}(e^{k_{1}t}-1)+k_{1}e^{k_{1}t}}\right)}^{2}$$

where *k*_1_ is the first‐order rate constant, defined as the inverse of the sum of electron and hole lifetimes, 1/(τ_n_ + τ_p_), and ${k^{\prime }}_{2}$ is the sum of non‐radiative $\left(k_{2}^{NR}\right)$ and external radiative $\left(k_{2}^{Ext}\right)$ bimolecular rates, respectively, given by:

(3) $${k^{\prime }}_{2}=k_{2}^{NR}+k_{2}^{Ext}=k_{2}^{NR}+k_{2}^{Int}\gamma$$

Here, the external radiative contribution is the product of the internal radiative bimolecular rate constant $k_{2}^{Int}$ and the escape probability γ.^[^ ^36^ ^]^ Using this model, the TRPL kinetics can be globally modeled across the full range of fluences for both the control and treated samples with just *k*_1_ and ${k^{\prime }}_{2}$ as fitting parameters (Figure S9 and Table S2, Supporting Information, for global model parameters). Importantly, we find that the differing evolution of the TRPL dynamics in each sample is controlled by the first‐order *k*_1_ parameter, while the bimolecular recombination parameter, ${k^{\prime }}_{2}$, remains relatively invariant. The fitted ${k^{\prime }}_{2}$ values vary by 1.3× the standard error, at (2.0 ± 0.8) × 10^−10^ cm^−3^ s^−1^ for the control sample and (0.95 ± 0.03) × 10^−10^ cm^−3^ s^−1^ for the treated sample. However, the extracted *k*_1_ decreases from (6.5 ± 0.3) × 10^6^ s^−1^ in the control to (0.25 ± 0.01) × 10^6^ s^−1^ in the sample post‐treated with an optimal BF_4_^−^ concentration (5 mg mL^−1^).

Figure 2b and Figure S9, Supporting Information, show that the kinetic model describes the experimental data well across the full range of excitation densities, with *k*_1_ controlling the drastically different dynamics of each sample. At high excitation density, the samples initially display second‐order behavior, as indicated by the decreasing slope of the PL decay with time. The control sample kinetics quickly become dominated by a first‐order process after ≈100 ns (see inset), owing to a *k*_1_constant which is 26× higher than in the treated sample. The contribution of Shockley–Read–Hall trap‐mediated recombination is therefore ≈1.5 orders of magnitude lower in the 5 mg mL^−1^ treated sample, indicating a substantial reduction in the density of trap states. This optoelectronic improvement is achieved with relatively limited alteration to the intrinsic second‐order recombination mechanisms of the materials.

Comparing the extracted rate constants from TRPL with our measurements of external PLQE, we calculate the relative contributions of radiative and non‐radiative bimolecular recombination in the control and 5 mg mL^−1^ treated samples (see Table S2, Supporting Information). Under solar illumination conditions the incident photon flux is 1.6 × 10^17^ photons cm^−2^, leading to a steady‐state carrier density that balances charge generation and recombination within the control sample of (8.0 ± 0.4) × 10^14^ cm^−3^. The treated sample maintains an excited‐state population of (63.2 ± 0.9) × 10^14^ cm^−3^ due to the minimization of non‐radiative loss pathways. For both samples, the measured external PLQE values under solar‐equivalent illumination point to a non‐radiative bimolecular contribution which is comparable to values previously communicated.^[^ ^41^, ^43^, ^44^ ^]^ By calculating an emitted photon escape probability of 8% for our MAPbI_3_ thin films (see Section S1, Supporting Information), we estimate that the internal PLQE increases from (5.7 ± 1.5)% to (59.9 ± 3.9)% upon post‐treatment. Even when including recent reports of photon recycling probabilities as high as 25%^[^ ^45^ ^]^ in MAPbI_3_ films of similar thickness, the internal PLQE values would be ≈2% and ≈32% in the control and treated films, respectively. We note that we do not see an appreciable effect of the treatment on higher‐order Auger recombination, which is primarily relevant at high excitation densities (Figure S10, Supporting Information). These spectroscopic and device observations are consistent with a large reduction in non‐radiative recombination with the treated samples.

### 2.2 Local Structural Determination

**Figure 3:**
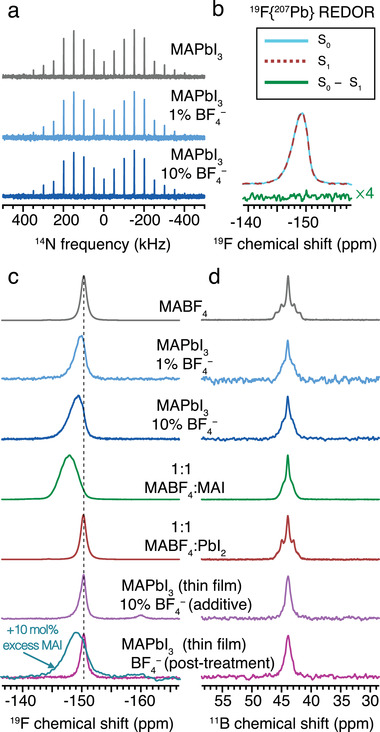
Solid‐state MAS NMR characterization of the materials. a) Hahn‐echo‐detected ^14^N spectra at 9.4 T, 298 K, and 5 kHz MAS of mechanochemical MAPbI_3_ and MAPbI_3_ with 1 and 10 mol% MABF_4_. b) ^19^F{^207^Pb} REDOR spectra of mechanochemical MAPbI_3_ with 10 mol% MABF_4_. c,d) Echo‐detected ^19^F (c) and ^11^B (d) NMR spectra at 7.0 T, 298 K, and 25 kHz MAS of neat MABF_4_, mechanochemical MAPbI_3_, MAPbI_3_ with 1 and 10 mol% MABF_4_, MABF_4_:MAI (1:1), MABF_4_:PbI_2_ (1:1) and thin films of MAPbI_3_ treated with 10 mol% MABF_4_ (as an additive to the precursor solution) or post‐treated with an IPA solution of MABF_4_ (10 mg mL^−1^). An additional ^19^F spectrum acquired on a thin film of MAPbI_3_ prepared with 10 mol% excess MAI and post‐treated with MABF_4_ is indicated by an arrow. The thin film samples were obtained by scraping the material off glass slides.

In order to establish the phase composition of BF_4_^−^‐treated MAPbI_3_ and verify whether or not BF_4_^−^ incorporates into the 3D perovskite lattice, we carried out ssNMR measurements on bulk microcrystalline mechanochemically synthesized perovskites,^[^ ^46^, ^47^ ^]^ as well as thin films (**Figure** **3**). We first look at bulk microcrystalline powders since they allow the exploration of BF_4_^−^ chemistry with high sensitivity through ssNMR. ^14^N NMR of the A‐site cation is a sensitive probe of the cubooctahedral symmetry in 3D perovskites, with narrower ^14^N spectral envelopes corresponding to higher lattice symmetry (closer to cubic).^[^ ^30^, ^31^, ^48^ ^]^ It has previously been shown that the incorporation of cesium into FAPbI_3_,^[^ ^30^ ^]^ MA^+^ into FAPbI_3_,^[^ ^48^ ^]^ and guanidinium into MAPbI_3_ and FAPbI_3_,^[^ ^31^ ^]^ leads to lowering of the cubooctahedral symmetry which translates to considerably broader ^14^N spinning sideband manifolds. Similar broadening has been observed in FAPbI_3_ treated with adamantylammonium iodide, which was explained in the context of structure directing effects.^[^ ^49^ ^]^ MAPbI_3_ with addition of 1 and 10 mol% of MABF_4_ yields ^14^N spectra which are essentially indistinguishable from that of untreated MAPbI_3_, strongly suggesting that the BF_4_^−^ anion is not incorporated into the bulk perovskite lattice as a pseudo halide ion (Figure 3a).

While ^14^N NMR probes the symmetry of the perovskite lattice, the local structure of the additive can be specifically probed using ^19^F and ^11^B NMR (Figure 3c,d). The ^19^F spectrum of the neat additive, MABF_4_, yields a single ^19^F resonance (δ = −150.3 ppm, full width at half maximum (fwhm) = 430 Hz), consistent with the presence of a single BF_4_^−^ environment inside the unit cell of MABF_4_.^[^ ^50^ ^]^ The ^19^F signal of BF_4_^−^ shifts to higher ppm values and broadens in MAPbI_3_ samples with 1 and 10 mol% MABF_4_ (δ = −149.6 ppm, fwhm = 587 Hz, and δ = −149.0 ppm, fwhm = 777 Hz, respectively), which indicates that the local environment has changed. However, a ^19^F{^207^Pb} rotational‐echo double‐resonance (REDOR)^[^ ^51^ ^]^ experiment (Figure 3b) corroborates the ^14^N data and shows that the BF_4_^−^ environment which gives rise to the ^19^F signal is not incorporated into the perovskite as a bulk dopant ion. In this REDOR experiment, dipolar coupling is reintroduced which would cause any ^19^F nuclei within ≈3.5 Å of ^207^Pb nuclei to dephase, reducing the signal intensity. However, the spectra with (S_1_) and without (S_0_) recoupling are identical (resulting in no observable signal in the difference spectrum, S_0_−S_1_). Therefore, there are no ^207^Pb nuclei in atomic‐level proximity of the BF_4_^−^, and we conclude that MAPbI_3_ and BF_4_^−^ are present in two separate phases.

Since there is no evidence of incorporation of the BF_4_^−^ into the perovskite, the change in local environment exhibited by the ^19^F spectrum could instead be caused by a side reaction between MABF_4_ and one or both of the precursors. Mechanochemically mixing equimolar amounts of MABF_4_ and MAI leads to a qualitatively similar downfield shift and broadening of the ^19^F signal of BF_4_^−^ (δ = −147.8, fwhm = 973 Hz), suggesting that these two materials form a cocrystal (Figure 3c). The XRD pattern of the 1:1 MABF_4_:MAI sample shows phase segregation into MAI and a MABF_4_‐rich cocrystal with lattice parameters that are intermediate between those of MABF_4_ and MAI (Figure S11 and Table S3, Supporting Information); this is the methylammonium mixed‐anion cocrystal phase observed by NMR, since all the BF_4_^−^ is present in this phase. Conversely, MABF_4_ does not react with PbI_2_, as evidenced by the unchanged ^19^F NMR signature in a 1:1 mechanochemical mixture of MABF_4_ and PbI_2_ (Figure 3c).

Having established the chemistry of BF_4_^−^ in bulk microcrystalline powders, we show that the BF_4_^−^ post‐treatment process used for the photophysics experiments also does not lead to incorporation of BF_4_^−^ into the perovskite; the ^19^F signal from a BF_4_^−^ post‐treated MAPbI_3_ film is essentially indistinguishable from neat MABF_4_ (Figure 3c, “post‐treatment”). We instead hypothesize that the atomic‐level mechanism of action of MABF_4_, leading to improved optoelectronic properties, is the binding of traces of residual unreacted MAI. This does not result in a significant change in the ^19^F signal since the amount of trace residual MAI, and hence the amount of MAI in the cocrystal, is so low. However, by preparing a thin film of MAPbI_3_ with 10 mol% excess MAI then post‐treating with MABF_4_ as above, the corresponding ^19^F spectrum clearly shows the formation of MAI–MABF_4_ cocrystals (bottom row, Figure 3c), thereby confirming that MABF_4_ is effective in scavenging residual MAI. Treating the MAPbI_3_ thin‐film with MABF_4_ via the DMSO precursor solution, on the other hand, again results in a ^19^F signal which is indistinguishable from the precursor (Figure 3c, “additive”); we ascribe the lack of substantial MAI–MABF_4_ cocrystal formation in this case to the strong dependence of cocrystallization on the presence of solvents.^[^ ^52^ ^]^

The ^11^B MAS NMR spectra (Figure 3d) provide complementary confirmation of the BF_4_^−^ chemistry, although these measurements are less sensitive to structural changes compared to the ^19^F spectra. The ^11^B spectrum of neat MABF_4_ exhibits a single resonance (δ = 43.9 ppm) showing scalar (*J*) coupling of 95 Hz to the four directly bonded ^19^F nuclei, leading to a quintet. In the perovskite samples prepared by mechanosynthesis, as well as in the equimolar MAI–MABF_4_ cocrystal, the scalar coupling is unresolved, with *J* ≈ 80 Hz, indicating a structural change. For the equimolar mixture of PbI_2_ and MABF_4_, in contrast, the *J*‐coupling pattern remains resolved with *J* = 90 Hz, corroborating the lack of reactivity between these two materials. Finally, for the ^11^B spectra of the treated thin films, no scalar coupling can be resolved within the experimental linewidth (≈50 Hz half‐width at half‐maximum). This is most likely due to solvent effects or partial hydration of the films, which were handled in air, since a similar spectrum was observed for MABF_4_ after storing in air (Figure S12, Supporting Information), and the ^11^B–^19^F coupling in BF_4_^−^ depends strongly on the solvent and the presence of hydrogen bonding.^[^ ^53^ ^]^

Taken together, the solid‐state NMR experiments show that BF_4_^−^ has no propensity to incorporate into the perovskite lattice of MAPbI_3_, and the results suggest that the optoelectronic improvements observed upon addition of MABF_4_ are due to the capacity of the additive to scavenge trace residual MAI and bind it in the form of MAI–MABF_4_ cocrystals. The unchanged PL emission spectra in treated samples is fully consistent with the lack of incorporation of BF_4_^−^ into the perovskite lattice.

**Figure 4:**
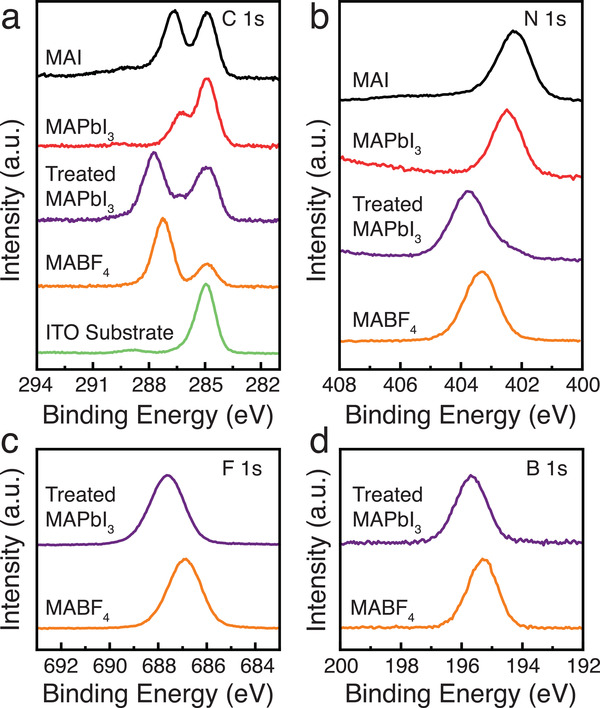
a–d) C 1s (C—N) (a), N 1s (b), F 1s (c), and B 1s (d) XPS spectra of control and post‐treated (10 mg mL^−1^ MABF_4_) MAPbI_3_ perovskite films, as well as neat MAI and MABF_4_ films. The intensities are scaled to similar values and vertically offset for clarity.

To complement the NMR spectroscopy, X‐ray photoelectron spectroscopy (XPS measurements (C 1s, N 1s, F 1s, and B 1s) were performed for pristine and post‐treated (10 mg mL^−1^) MAPbI_3_ perovskite films to obtain surface‐selective local structural information on the perovskite and the MABF_4_ additive (**Figure** **4**). The C 1s and N 1s signals associated with MA environments in MAPbI_3_ (286.27, 402.43 eV) and MABF_4_ (287.70, 403.70 eV) are well resolved. All C 1s spectra also contain a well‐resolved signal at 284.92 eV originating from C—C bonds in spurious trace organic impurities typically detected in XPS,^[^ ^54^ ^]^ which are also present in an untreated ITO substrate. The C 1s, N 1s, F 1s, and B 1s peak positions associated with MABF_4_ are shifted in MABF_4_‐treated MAPbI_3_ compared to neat MABF_4_, which is consistent with the formation of MAI–MABF_4_ cocrystals evidenced by NMR. Specifically, we observe the following changes in binding energies: C 1s (286.27 vs 287.70 eV), N 1s (402.43 vs 403.70 eV), F 1s (686.88 vs 687.64 eV) and B 1s (195.22 vs 195.68 eV). Moreover, we do not observe significant changes in the shape of the I 3d and Pb 4f spectra (Figure S13, Supporting Information), which confirms that there is no detectable change in the MAPbI_3_ structure or chemical composition, in agreement with the NMR data. Since XPS measurements are surface‐sensitive, we conclude that the formation of the MAI–MABF_4_ cocrystal proceeds spontaneously upon spin‐coating and the cocrystal is present at least on the surface of the film. The interaction of MABF_4_ with the film is driven by the presence of local excess MAI, which is effectively scavenged and bound as a MAI–MABF_4_ cocrystal.

Numerous computational studies have shown that the defects responsible for deep trap states that lead to non‐radiative recombination are related to interstitial iodide or lead vacancies, both of which result in undercoordinated iodide.^[^ ^55^, ^56^ ^]^ Experimentally, it has been found that strategies aimed at removing these types of defects effectively remove deep traps in MAPbI_3_.^[^ ^57^, ^58^ ^]^ The source of excess iodide in the system at hand may be trace MAI present in the film after deposition. The solid‐state NMR and XPS analyses unambiguously show that MABF_4_ acts as a MAI scavenger, thereby contributing to the removal of excess iodide from the film surfaces. Finally, the solid‐state NMR results provide clear evidence that there is no atomic‐level interaction between BF_4_^−^ and undercoordinated lead in the perovskite structure, suggesting that the optoelectronic improvements here do not relate to the passivation of these sites. This mechanism of action is reminiscent of that previously reported for potassium addition into halide perovskites. While solid‐state NMR has unambiguously shown that there is no potassium incorporation into 3D halide perovskites,^[^ ^32^ ^]^ a considerable reduction in non‐radiative losses has been reported in KI‐treated hybrid perovskite thin films.^[^ ^14^ ^]^ We hypothesize that this type of halide management may be the main mechanism of action for small inorganic ions which do not incorporate into the perovskite lattice but rather phase‐segregate to form secondary phases.^[^ ^30^ ^]^ Other examples of dopants exhibiting similar phase segregation behavior include Ba^2+^ and Co^2+^.^[^ ^59^, ^60^ ^]^ Thus, our work provides general mechanistic understanding: these approaches ultimately equate to different forms of halide (or halide vacancy) management, including facilitating fine‐tuning of the halide composition so as to reduce defect densities and non‐radiative charge recombination pathways, thereby leading to enhanced device performance (Figure S7 and Table S1, Supporting Information).

## 3 Conclusion

We have studied the effects on the optoelectronic properties of introducing an organic tetrafluoroborate salt to the surface of hybrid perovskite MAPbI_3_ thin films in the form of a post‐treatment. Measurements of the PLQE and PL lifetimes of treated samples show an increased prominence of radiative recombination pathways. The most effective treatment results in hybrid perovskite MAPbI_3_ films with an external PLQE of 10.4% and a monomolecular PL lifetime at low fluence of 952 ± 23 ns. By modeling the fluence‐dependent kinetics, we show that the optimized BF_4_^−^ treatment reduces the deep trap density by a factor of 26 without significant alteration of the intrinsic radiative properties of the MAPbI_3_ material. Furthermore, we have shown by NMR spectroscopy that BF_4_^−^ is not incorporated into the perovskite, but instead participates in cocrystal formation in a reaction between MABF_4_ and MAI. The removal of trace unreacted MAI, driven by the thermodynamic stability of the MAI–MABF_4_ cocrystal, decreases the concentration of interstitial iodide defects in the perovskite, which otherwise act as deep traps, and thus substantially decreases non‐radiative recombination. This analysis sheds light on the defect density reduction mechanism, and also the reaction dynamics with non‐essential entities in hybrid perovskites. Such halide management strategies, through formation of new, benign cocrystals without changing the chemical structure or optical bandgap of the hybrid perovskite, provide an exciting, generalized template for further defect removal approaches.

## 4 Experimental Section

### Perovskite Film Synthesis and Fabrication

PbI_2_ (1 m) and MAI (1 m) were dissolved in 4:1 DMF:DMSO under continuous stirring and heating at 70 °C for 30 min. The perovskite solution was then spin coated (4000 rpm for 40 s) on plasma cleaned glass substrates. Perovskite films were annealed at 100 °C for 1 h. Annealed films were allowed to cool to room temperature and then a solution of MABF_4_ in IPA was spin coated on the top of the perovskite films (for control neat IPA was used in post‐treatment). All these processes were carried out in a nitrogen glove box. Detailed device fabrication methods are provided in the Supporting Information.

### Perovskite Mechanosynthesis

The starting materials were stored inside a glove box under argon. MABF_4_ was thoroughly dried at 80 °C under vacuum overnight. Perovskite powders were synthesized by grinding the reactants in an electric ball mill (Retsch Ball Mill MM‐400) using an Eppendorf vial (2 mL) and a stainless‐steel ball (⌀4 mm) for 30 min at 25 Hz. The resulting powders were annealed for 5 min at 120 °C to remove grinding‐induced defects. The reagent quantities used in the syntheses are given in the Supporting Information.

### Optical Spectroscopy

UV–vis absorption of thin films was recorded on an Agilent 8453 UV–vis spectrophotometer with deuterium (190–800 nm) and tungsten (310–1100 nm) lamps. The spectrometer is equipped with a photodiode array for detection. PL of thin films was measured on an Edinburgh Instruments FLS980 fluorimeter. PL measurements were performed with a 470 nm excitation wavelength. PLQY measurements were performed with a 520 nm continuous‐wave diode laser with an excitation density of ≈60 mW cm^−2^ in an integrating sphere. The emission of samples was measured using an Andor iDus Si detector. This detector was calibrated with a HL‐3P‐CAL Ocean optics broadband source. XPS measurements were performed by a monochromatic Al Kα X‐ray source (hν = 1486.6 eV) using a SPECS PHOIBOS 150 electron energy analyzers with a total energy resolution of 500 meV. Conductive silver paint was used to connect the sample surface to the holder to avoid charge accumulation.

### TRPL

TRPL measurements were carried out using a 407 nm pulsed supercontinuum laser as an excitation source. An absorptive 420 nm long‐pass filter was used to filter out scattered laser light. The focused PL was detected by a silicon‐based single photon avalanche photodiode (MPD‐PDM‐PDF). Instrument response time was ≈200 ps.

For the investigation of recombination behavior, time‐resolved PL spectra were recorded using a gated intensified CCD camera (Andor iStar DH740 CCI‐010) connected to a calibrated grating spectrometer (Andor SR303i). The 800 nm emissions from a Ti:sapphire optical amplifier (1 kHz repetition rate, 90 fs pulse width) was frequency‐doubled to generate narrow bandwidth excitation centered at a wavelength of 400 nm. The incident pulse energy was varied from 0.0152–1.52 µJ cm^−2^. Initial excited carrier densities were calculated according to the method of Richter et al.^[^ ^41^ ^]^ The effective area of the excitation spot was 1.48 mm^2^.

### XRD

XRD measurements of perovskite thin films were performed using a Bruker X‐Ray D8 Advance diffractometer, while XRD measurements of mechanosynthesized powders were performed with a PANalytical Empyrean diffractometer. Both diffractometers used Cu Kα radiation (λ = 1.54 Å).

### Solid‐State NMR

Room temperature ^19^F (282.5 MHz) and ^11^B (96.3 MHz) MAS NMR spectra were recorded on a Bruker Avance III 7.0 T spectrometer equipped with a 2.5 mm CPMAS probe. ^14^N (28.9 MHz) MAS NMR spectra were recorded on a Bruker Avance 9.4 T spectrometer equipped with a 4.0 mm CPMAS probe. ^19^F chemical shifts were referenced to CFCl_3_ using C_6_F_6_ (δ = −164.9 ppm) as a secondary reference. ^11^B shifts were referenced to 15% BF_3_.OEt_2_ using NaBH_4_ (δ = 3.2 ppm) as a secondary reference. ^207^Pb chemical shifts were referenced to Me_4_Pb using Pb(NO_3_)_2_ (δ = −3494 ppm at room temperature) as a secondary reference. The REDOR experiments used 5 rotor periods of recoupling. Further experimental details and acquisition parameters can be found in Tables S4–S7, Supporting Information.

## Conflict of Interest

S.D.S. is a co‐founder of Swift Solar, Inc. All other authors declare no conflict of interest.

## Supporting information

Supporting Information

## Data Availability

The data that support the findings of this study are openly available in the Cambridge Research Repository Apollo at https://doi.org/10.17863/CAM.70304.
