# Supplementary material for: Tetrafluoroborate‐Induced Reduction in Defect Density in Hybrid Perovskites through Halide Management
Source: Adv Mater. 2021 Jul 4;33(32):2102462. doi: 10.1002/adma.202102462 (PMC11468984; doi:10.1002/adma.202102462)
Supplement: Supplementary file 1 — Supporting Information [file ADMA-33-2102462-s001.pdf]

© 2021 Wiley-VCH GmbH

ADVANCED MATERIALS

Supporting Information

for *Adv. Mater.,* DOI: 10.1002/adma.202102462

Tetrafluoroborate-Induced Reduction in Defect Density in Hybrid Perovskites through Halide Management

*Satyawan Nagane, Stuart Macpherson, Michael A. Hope, Dominik J. Kubicki, Weiwei Li, Sachin Dev Verma, Jordi Ferrer Orri, Yu-Hsien Chiang, Judith L. MacManus-Driscoll, Clare P. Grey, and Samuel D. Stranks**

© Copyright 2021 WILEY-VCH GmbH.

Supporting Information

Tetrafluoroborate-Induced Reduction in Defect Density in Hybrid Perovskites through Halide Management

*Satyawan Nagane, Stuart Macpherson, Michael A. Hope, Dominik J. Kubicki, Weiwei Li, Sachin Dev Verma, Jordi Ferrer Orri, Yu-Hsien Chiang, Judith L. MacManus-Driscoll, Clare P. Grey, Samuel D. Stranks**

Fabrication of triple cation-based perovskite solar cell devices:

Fabrication of SAMs:

ITO coated glasses (Kintec) were cleaned by ultra-sonication in four cycles, first cycle was water with Hellmanex™ for 15 minutes followed by cleaning with fresh water (sonication for 5 minutes). The water-cleaned substrates were then ultra-sonicated for 15 minutes in Acetone and finally in isopropanol for 15 minutes. These conducting glass substrates were dried using a nitrogen gun. The dried substrates were then cleaned by UV-Ozone plasma cleaner for 15 minutes. To fabricate the self-assembled monolayers (SAMs) on plasma cleaned substrate, we transferred these substrates to nitrogen glovebox. SAMs used here was **2PACz** ([2-(9*H*-carbazol-9-yl)ethyl]phosphonic acid).[1] SAMs were dissolved in anhydrous Ethanol. The concentration used was 1mmol/L (~3 mg/mL for 2PACz). The SAMs solution (50 µL) were dropped onto the ITO substrates and spin coated at 2000 rpm for 40 seconds. The substrates were then heated at 100°C for 10 minutes. The SAMs containing substrates were then cooled down to room temperature and used for fabrication of perovskite layer.

Fabrication of $MAPbI_3$-based solar cell devices:

PbI_2 (0.277 g, 1.2 mmol) and MAI (0.095 g, 1.2 mmol) were dissolved in DMF:DMSO (4:1) at room temperature under continuous stirring. The perovskite solution was then spread on HTL containing conducting glass and spin coated at 3000 rpm for 30 seconds. An antisolvent (chlorobenzene) was dispensed on perovskite film 10 seconds before the end of spinning programme. The perovskite films were then annealed at 65 ^{o}C for 10 minutes and 100 ^{o}C for 10 minutes. The $MABF_4$ (1 mg/mL in isopropanol) was spin coated on the surface of annealed films, and neat isopropanol on the control films. After treatment, 20 nm of C_{60} and 6 nm of BCP were thermally evaporated on control as well as treated samples. Subsequently, silver (100 nm) was deposited by thermal evaporation.

Fabrication of triple cation perovskite solar cell devices:

The triple-cation perovskite [$Cs_{0.05}(MA_{0.17}FA_{0.83})_{0.95}Pb(I_{0.83}Br_{0.17})_3$] solution was prepared by dissolving PbI_2 (253.56 mg, 1.1 mmol), FAI (85.98, 1mmol), $PbBr_2$ (40 mg, 0.2 mmol) and MABr (12.15 mg, 0.2 mmol) in a mixture of solvents consisting of dimethyl sulfoxide (170 µL), dimethyl formamide (75 µL) and γ-butyrolactone (255 µL) under continuous stirring at 70oC for 30 minutes. CsI (194.86 mg in 0.5 mL) was separately dissolved in dimethyl sulfoxide by heating at 70 ^{o}C under continuous stirring. 24 µL of CsI was then added in to the perovskite solution and the whole mixture again heated at 70°C for 30 minutes under continuous stirring. The perovskite solution (40 µL) was then cast on the SAMs containing conducting glass and spin coated at 1000 rpm for 10 seconds and 6000 rpm for 25 seconds. The antisolvent chlorobenzene was dispensed on the film 10 seconds before the end of spinning programme. The perovskite films were then annealed at 65°C for 10 minutes and then 100°C for 30 minutes. The $MABF_4$ (1 mg/mL in isopropanol) was spin coated on the surface of annealed films, and neat isopropanol on the control films. After treatment of $MABF_4$, 20 nm of C_{60} and 6 nm of BCP were thermally evapoarted on control as well as treated samples. Subsequently, silver (100 nm) was deposited by thermal evaporation.

Extracting recombination parameters from steady-state and time-resolved photoluminescence measurements:

Using the fitted rate constants, k_1 and $k_2{'}$, we can calculate the photoexcited carrier density under steady-state illumination by balancing the generation (G) and recombination rates. Excluding the role of 3rd order recombination processes, we have:

$$G - k_1 n - k_2{'} n^2 = 0$$

Solving this quadratic equation yields the solution for the steady state carrier density:

$$n = \frac{-k_1 + \sqrt{k_1^2 + 4k_2{'}G}}{2k_2{'}}$$

Setting G equal to the generation rate under solar equivalent illumination conditions (irradiance of ~60 mW/cm^2 at an excitation wavelength of 532 nm, providing an incident photon flux of ~1.6 × 10^{17} photons/cm^2) we calculate the steady-state carrier density, n^{Sun}, for our 300 nm thick films. The resulting values are displayed in Table S1.

The non-radiative and external radiative bimolecular rate constants, k_2^{NR} and k_2^{Ext} can be decoupled by comparison of measured external PLQE values, with theory:

$$PLQE^{Ext} = \frac{k_2^{Ext} n}{k_1 + k_2{'} n} = \frac{k_2^{Ext} n}{k_1 + (k_2^{Ext} + k_2^{NR}) n}$$

The resulting values for each sample are displayed in Table S1.

As outlined in the main text, the external radiative bimolecular recombination rate is the product of the internal rate (k_2^{Int}) and the escape probability(γ). An estimate for the escape probability for our planar $MAPbI_3$ thin films on glass, can be made using a simplified approach which has been previously applied to solution processed $MAPbI_3$ and GaAs thin films.[2,3] This is also published in Table S1. We approximate the escape probability through either interface as $2 \times \frac{1}{4n_r^2} \approx 8.0$ %, where the refractive index at the band edge (n_r) is ~2.5, according to ellipsometry measurements by Phillips et al.[4]

Mechanosynthesis

$MAPbI_3$ doped with 1 mol% $MABF_4$: 0.159 g MAI (1.00 mmol), 0.461 g PbI_2 (1.00 mmol), 0.001 g $MABF_4$ (0.01 mmol).

$MAPbI_3$ doped with 10 mol% $MABF_4$: 0.159 g MAI (1.00 mmol), 0.461 g PbI_2 (1.00 mmol), 0.012 g $MABF_4$ (0.10 mmol).

MAI:$MABF_4$ (1:1): 0.159 g MAI (1.00 mmol), 0.119 g $MABF_4$ (1.00 mmol).

PbI_2:$MABF_4$ (1:1): 0.461 g PbI_2 (1.00 mmol), 0.119 g $MABF_4$ (1.00 mmol).

Methods:

Transient absorption spectroscopy

Fluence-dependent transient absorption spectroscopy measurements were conducted at picosecond timescales. This pump-probe technique is used to track the differential transmission in thin film samples induced by a photoexcited carrier population.
Optical pulses were generated with a PHAROS (Light Conversion) laser (38 kHz repetition rate, 200 fs pulse duration). The probe pulse was a chirped white-light continuum generated by focusing the fundamental (1030 nm) in a YAG crystal. The 550 nm pump pulse was generated in an ORPHEUS (Light Conversion) optical parametric amplifier. The optical pulses were spatially overlapped and temporally delayed using a piezoelectric delay stage. The transmitted probe pulses were guided to a spectrometer with a Si photodiode array detector. Initial photoexcited carrier density (n_0) is calculated from $\Delta T/T$ using the incident pump spot geometry on the sample and adopting the method of Richter et al.[5]

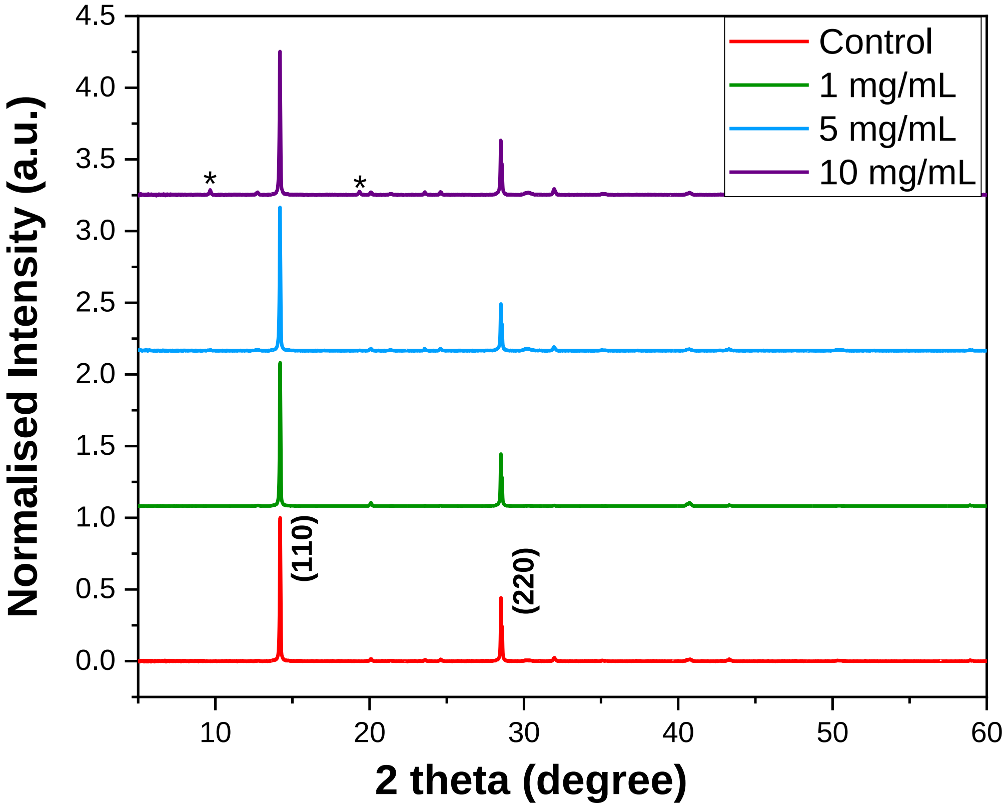

Figure S1. XRD pattern of $MAPbI_3$ (control) perovskite and $MAPbI_3$ perovskite treated with $MABF_4$ (1 mg/mL, 5 mg/mL and 10 mg/mL) dissolved in isopropanol (* denotes unreacted $MABF_4$ and/or MAI-$MABF_4$ cocrystals).

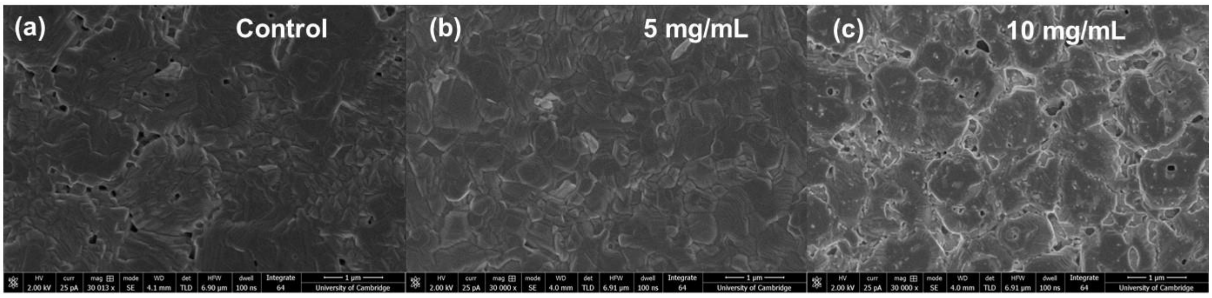

Figure S2. SEM images of $MAPbI_3$ (control) perovskite and $MAPbI_3$ perovskite treated with $MABF_4$ (5 mg/mL and 10 mg/mL) dissolved in isopropanol.

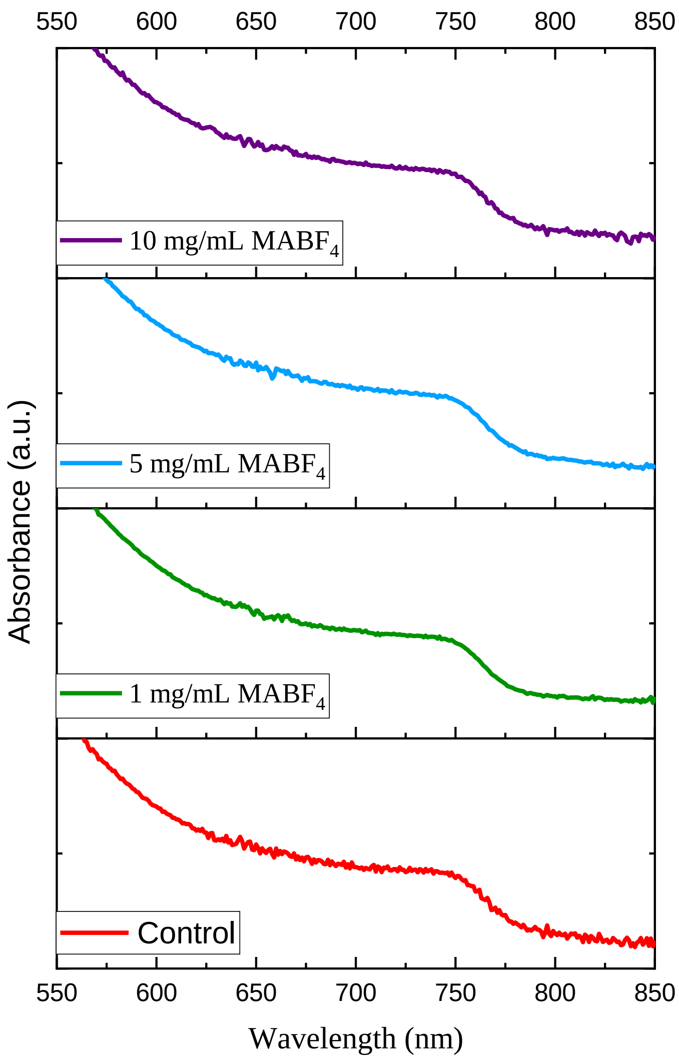

Figure S3. UV-Vis absorption spectra of $MAPbI_3$ (control) perovskite and $MAPbI_3$ perovskite treated with $MABF_4$ (1 mg/mL, 5 mg/mL and 10 mg/mL) dissolved in isopropanol.

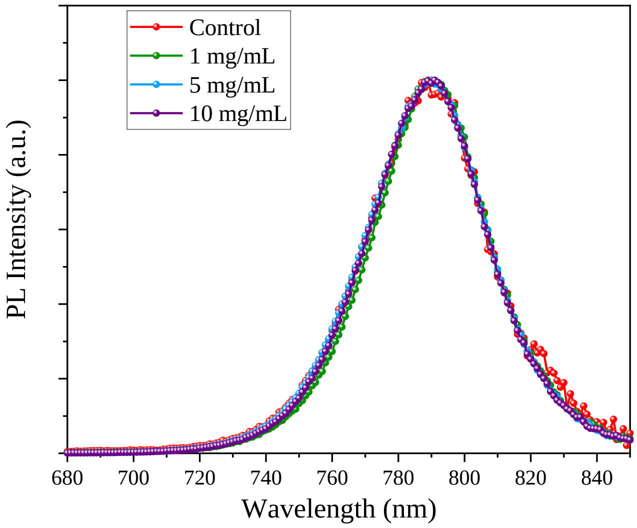

Figure S4. Normalized PL spectra $MAPbI_3$ (control) perovskite and $MAPbI_3$ perovskite treated with $MABF_4$ (1 mg/mL, 5 mg/mL and 10 mg/mL) dissolved in isopropanol.

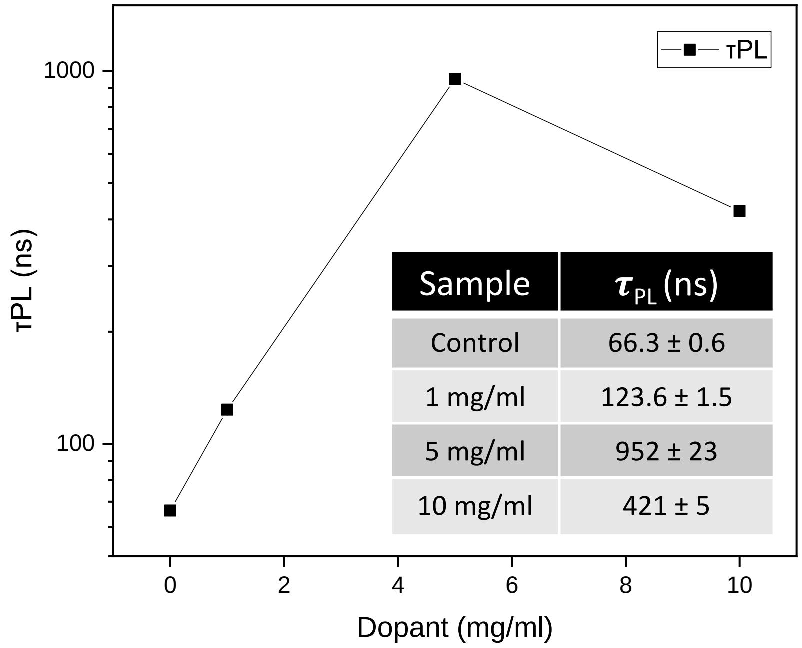

Figure S5. Extracted mono-exponential PL lifetimes of control and BF_4 treated $MAPbI_3$ perovskite samples, from TRPL data (Figure 1C).

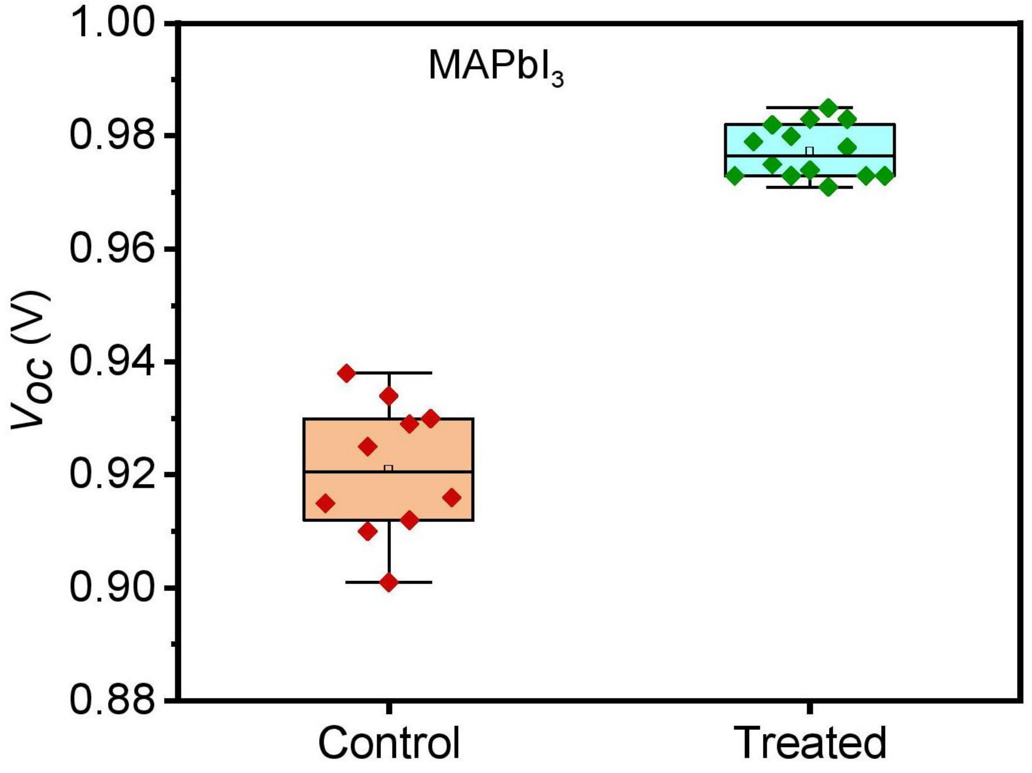

Figure S6. Distribution of device open-circuit voltages (V_{oc}) of $MAPbI_3$-based solar cells

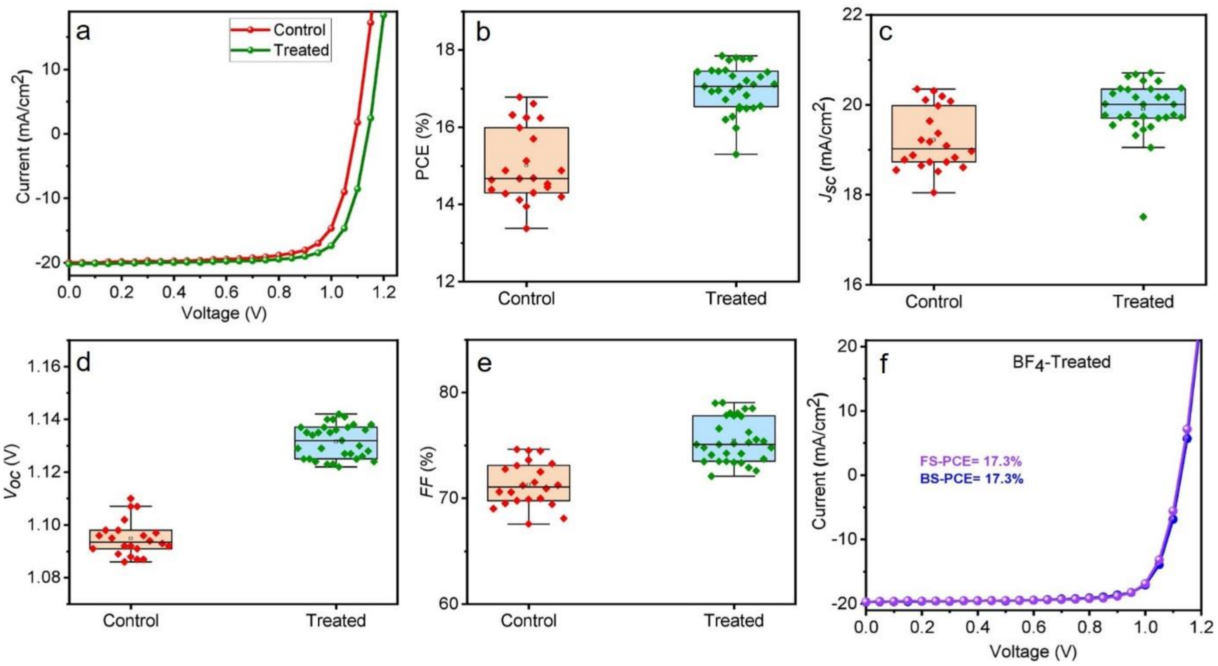

Figure S7. (a) *J-V* characteristics of representative solar cell devices employing the $Cs_{0.05}(MA_{0.17}FA_{0.83})_{0.95}Pb(I_{0.83}Br_{0.17})_3$ absorber layer without treatment (Control) and layers treated with $MABF_4$ (1 mg/mL, Treated). We note that 1 mg/mL treatment was optimal for devices. The corresponding device parameters are shown in Table S2. (b – e) Box-and-whisker statistical results of device parameters (power-conversion efficiency (PCE), short-circuit current (J_{sc}), open-circuit voltage (V_{oc}), fill factor (FF)) from devices across 3 batches. (f) *J-V* characteristics for representative BF_4-treated (1 mg/mL) $Cs_{0.05}(MA_{0.17}FA_{0.83})_{0.95}Pb(I_{0.83}Br_{0.17})_3$-based devices showing forward scan (FS) and backward scan (BS) curves.

Table S1. Device parameters from the representative perovskite solar cells shown in Figure S10a.

**Devices**	J_{sc} **(mA/cm²)**	V_{oc} **(V)**	FF **(%)**	**PCE (%)**
**Control**	19.9	1.09	74.5	16.3
**Treated**	20.2	1.14	76.3	17.6

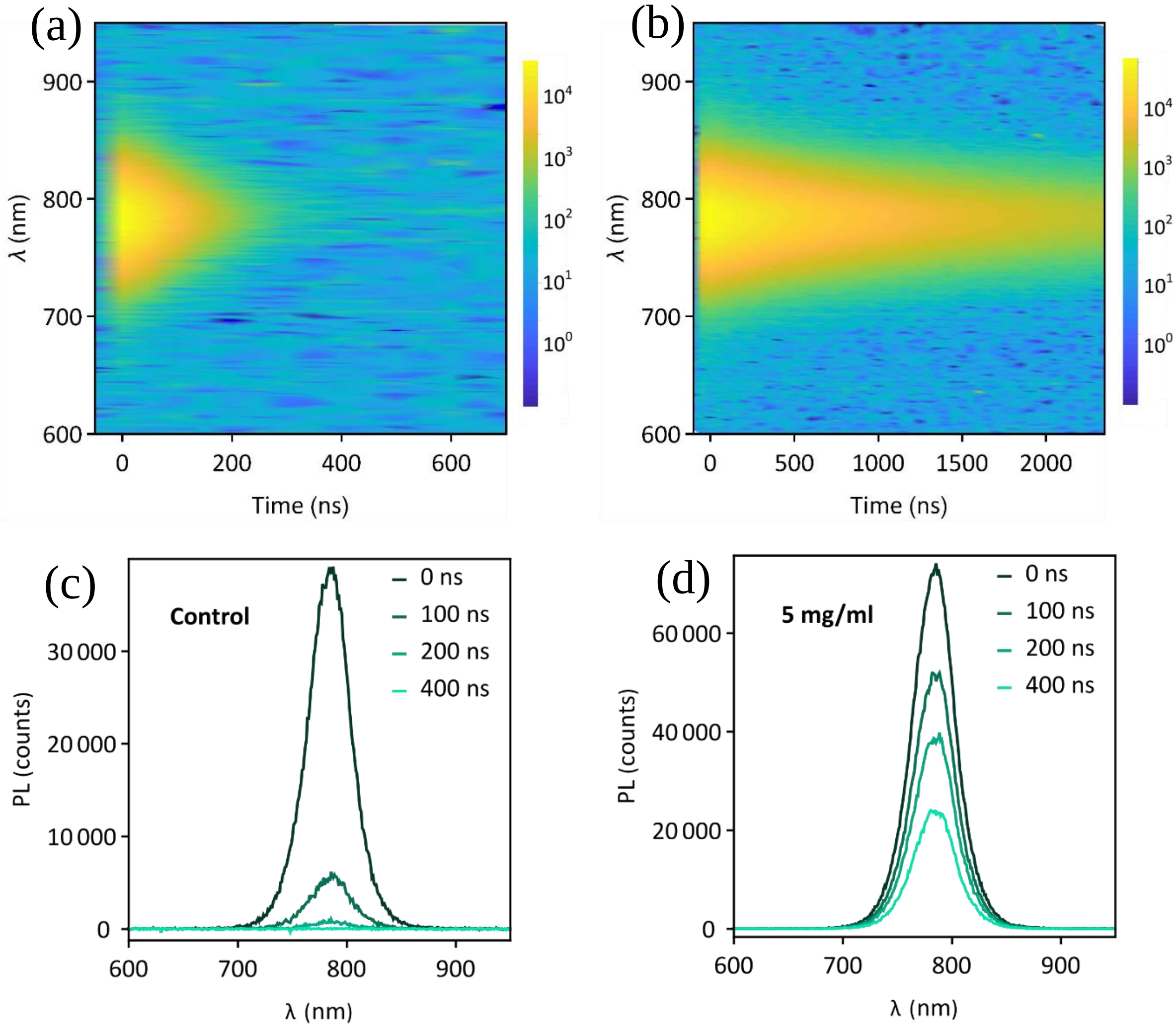

Figure S8: Time resolved PL spectra upon initial excitation density n_0 = 10^{16} cm^{-3}. Density plots of the PL evolution for the (a) control and (b) 5 mg/ml treated samples. (c) & (d) PL spectra at labeled time slices, for the respective samples.

Sample	Control	5 mg/ml
$k_1\ (s^{-1})$	(6.5 ± 0.3) x 10^{6}	(0.25 ± 0.01) x 10^{6}
$k_2'\ (cm^3 s^{-1})$	(2.0 ± 0.8) x 10^{-10}	(0.95 ± 0.03) x 10^{-10}
$C\ (cts^{1/2} cm^3)$	(1.3 ± 0.3) x 10^{-14}	(1.64 ± 0.02) x 10^{-14}
$n^{Sun}\ (cm^{-3})$	(8.0 ± 0.4) x 10^{14}	(63.2 ± 0.9) x 10^{14}
$PLQE^{Ext}$ (%)	0.50	10.42
$k_2^{NR}\ (cm^3 s^{-1})$	(1.6 ± 0.8) x 10^{-10}	(0.81 ± 0.03) x 10^{-10}
$k_2^{Ext}\ (cm^3 s^{-1})$	(0.4 ± 0.1) x 10^{-10}	(0.14 ± 0.01) x 10^{-10}
$k_2^{Int}\ (cm^3 s^{-1})$ $\gamma = 0.08$	(5.0 ± 1.3) x 10^{-10}	(1.8 ± 0.1) x 10^{-10}
$PLQE^{Int}$ (%)	(5.7 ± 1.5)	(59.9 ± 3.9)

Table S2: Fitting parameters for model specified by equation 2, for both the control and 5 mg/ml treated sample. C^2 is the constant of proportionality between the time-dependent photoexcited carrier density and photoluminescence. The carrier density under CW solar illumination conditions (n^{Sun}) is used to extract the non-radiative (k_2^{NR}) and external radiative (k_2^{Ext}) bimolecular rate constants. The calculated internal radiative (k_2^{Int}) bimolecular rate constant is provided for the treated sample, based on an estimated escape probability of $\gamma =$ 8.0 % for our $MAPbI_3$ thin films on glass. The escape probability was calculated as outlined in supplementary section 1.

For the control and treated samples respectively, 76% (61-89%) and 69% (67-71%) of internal bimolecular processes are radiative recombination events, as determined by the relative magnitudes of the non-radiative and internal radiative coefficients.

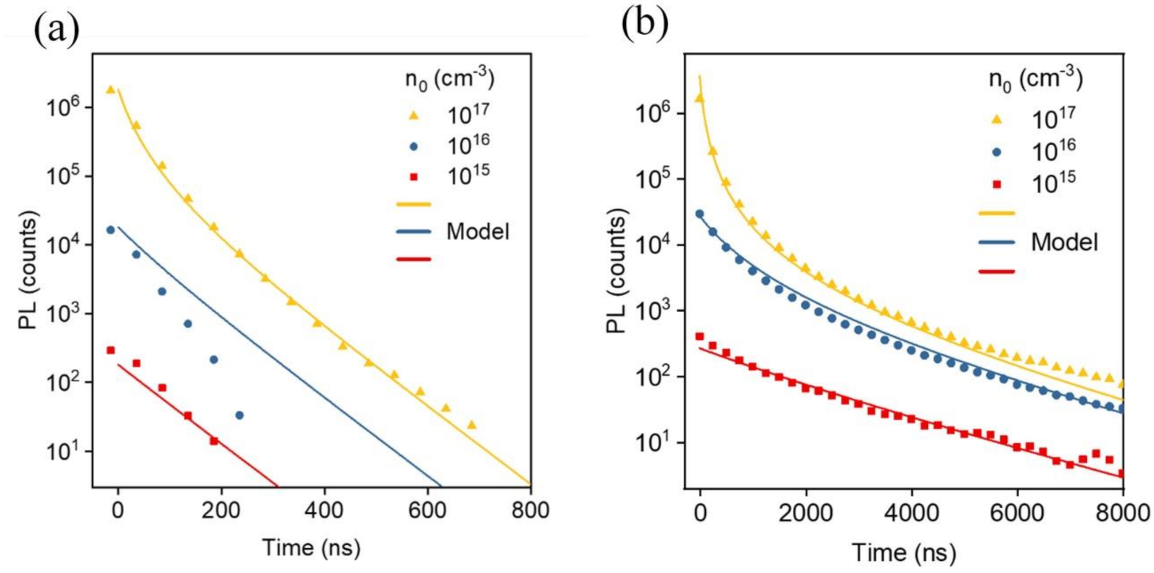

Figure S9: Spectrally integrated TRPL kinetics for (a) control and (b) 5 mg/ml samples across a range of initial excitation densities (n_0). Solid lines indicate simulated TRPL decays based on the model outlined in equation 2 of the main text and the fitted parameters expressed in Table S1.

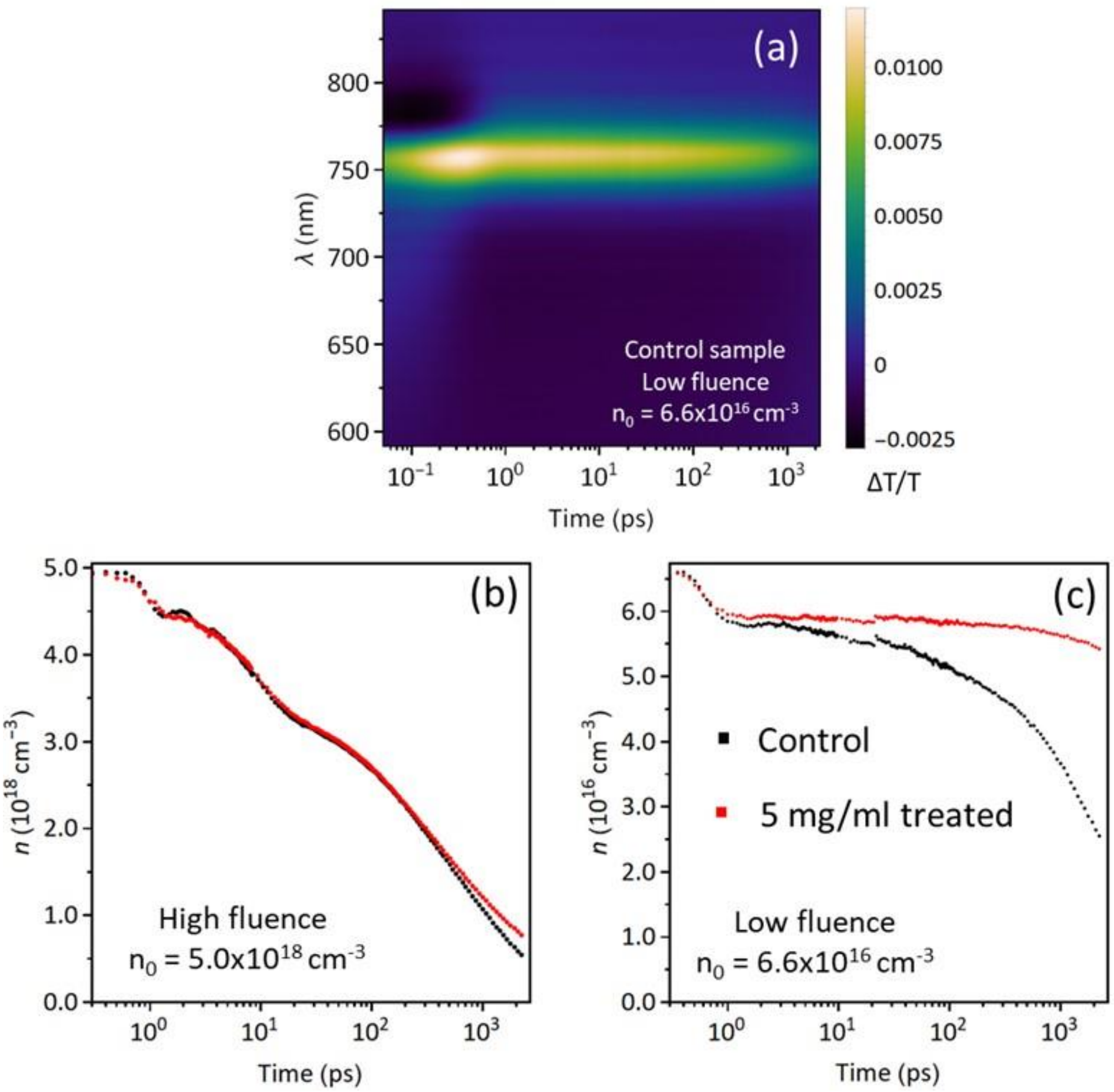

Figure S10: Fluence-dependent transient absorption spectroscopy measurements of the control and 5 mg/ml BF_4 treated $MAPbI_3$. (a) Example differential transmission spectrum of control sample with an initial excited carrier density of n_0 = 6.6x10^{16} cm^{-3}. Spectrally-integrated ground state bleach kinetics (744-770 nm) for the control and 5 mg/ml BF4 treated samples with an initial carrier density of (b) n_0 = 5.0x10^{18} cm^{-3} and (c) n_0 = 6.6x10^{16} cm^{-3}.

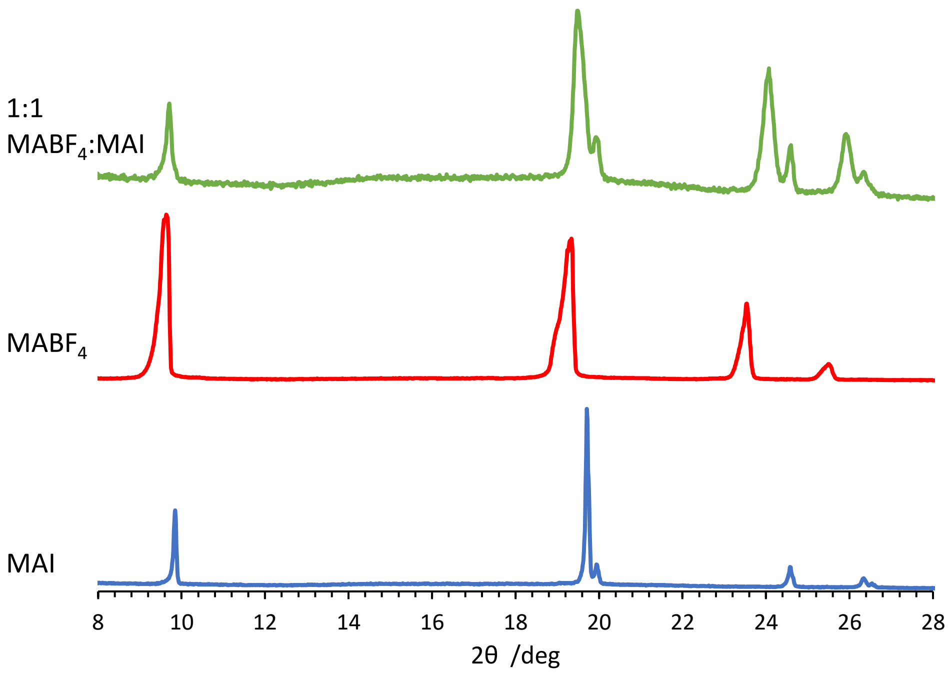

Figure S11: Selected region of the powder XRD patterns for $MABF_4$, MAI and a 1:1 ball-milled mixture.

Table S3: Lattice parameters (*P*4/*nmm*) from Pawley refinement of $MABF_4$, MAI and ball-milled 1:1 $MABF_4$:MAI mixture. The $MABF_4$:MAI sample is phase segregated, forming a $MABF_4$ rich cocrystal and MAI. The structures were taken from CCDC entries 131427 and 1946189.

	**Pure**		**1:1 Mix**	
	$MABF_4$	MAI	$MABF_4$-rich cocrystal	MAI
a /Å	5.361	5.121	5.231	5.121
c /Å	9.215	9.009	9.123	9.023

Table S4. Experimental parameters for the ^{14}N spectra reported in the main text.

spectrum	^{14}N T_1 [s]	recycle delay [s]	number of scans
$MAPbI_3$	0.15 (measured at 11.7 T)	0.5	1200
$MAPbI_3$ + 1 mol% $MABF_4$	0.15 (measured at 11.7 T)	0.5	1200
$MAPbI_3$ + 10 mol% $MABF_4$	0.15 (measured at 11.7 T)	0.5	1200

Table S5. Experimental parameters for the ^{19}F spectra reported in the main text.

spectrum	^{19}F T_1 [s]	recycle delay [s]	number of scans
$MABF_4$	9.5	100	16
$MAPbI_3$ + 1 mol% $MABF_4$	-	100	16
$MAPbI_3$ + 10 mol% $MABF_4$	8.4	100	16
MAI + $MABF_4$ (1:1)	8.9	100	16
PbI_2 + $MABF_4$ (1:1)	-	100	16
$MAPbI_3$ + 10 mol% $MABF_4$ (thin film, additive)	5	25	32

$MAPbI_3$ + $MABF_4$ (thin film, passivation layer)	-	25	16
$MAPbI_3$ (10 mol% excess MAI) + $MABF_4$ (thin film, passivation layer)	-	5	1000

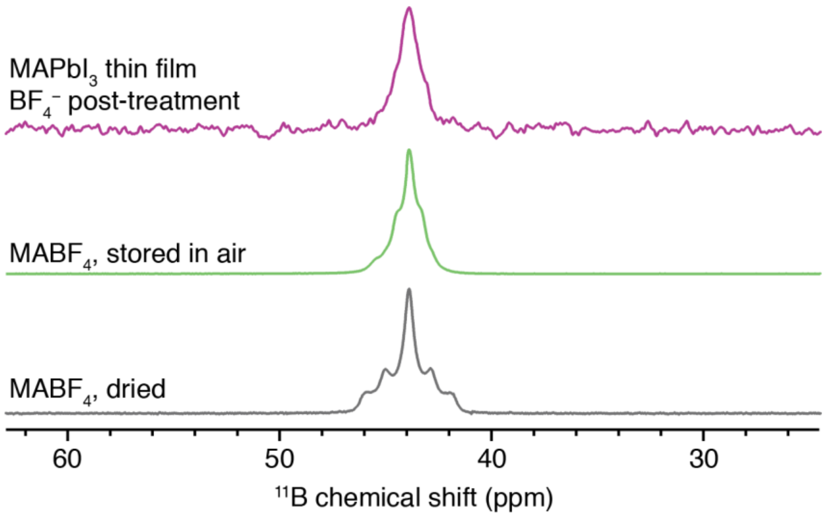

Figure S12: ^{11}B spectra of dried $MABF_4$, $MABF_4$ after storing in air, and a $MAPbI_3$ thin film post-treated with an isopropanol solution of $MABF_4$ (10 mg/mL). After storing in air, $MABF_4$ shows unresolved scalar coupling with $J \approx 50$ Hz, resulting in a similar overall linewidth to the post-treated thin film $MAPbI_3$ sample.

Table S6. Experimental parameters for the ^{11}B spectra reported in the main text.

spectrum	^{11}B T_1 [s]	recycle delay [s]	number of scans
$MABF_4$	4.2	100	16
$MAPbI_3$ + 1 mol% $MABF_4$	-	10	160
$MAPbI_3$ + 10 mol% $MABF_4$	-	100	32
MAI + $MABF_4$ (1:1)	-	10	160
PbI_2 + $MABF_4$ (1:1)	-	100	16
$MAPbI_3$ + 10 mol% $MABF_4$ (thin film, additive)	5	10	32
$MAPbI_3$ + $MABF_4$ (thin film, passivation layer)	-	10	64

Table S7. Shifts and peak widths of the spectra reported in figure 3 of the main text.

Material	^{19}F chemical shift [ppm]	^{19}F FWHM [Hz]	^{11}B chemical shift [ppm]
$MABF_4$	-150.3	317	43.9
$MAPbI_3$ + 1 mol% $MABF_4$	-149.6	587	43.9
$MAPbI_3$ + 10 mol% $MABF_4$	-149	777	43.9
MAI + $MABF_4$ (1:1)	-147.8	973	43.9
PbI_2 + $MABF_4$ (1:1)	-150.3	313	43.9

$MAPbI_3$ + 10 mol% $MABF_4$ (thin film, additive)	-150.3	321	43.9
$MAPbI_3$ + $MABF_4$ (thin film, passivation layer)	-150.3	328	43.9
$MAPbI_3$ (10 mol% excess MAI) + $MABF_4$ (thin film, passivation layer)	-149.0	1026	-

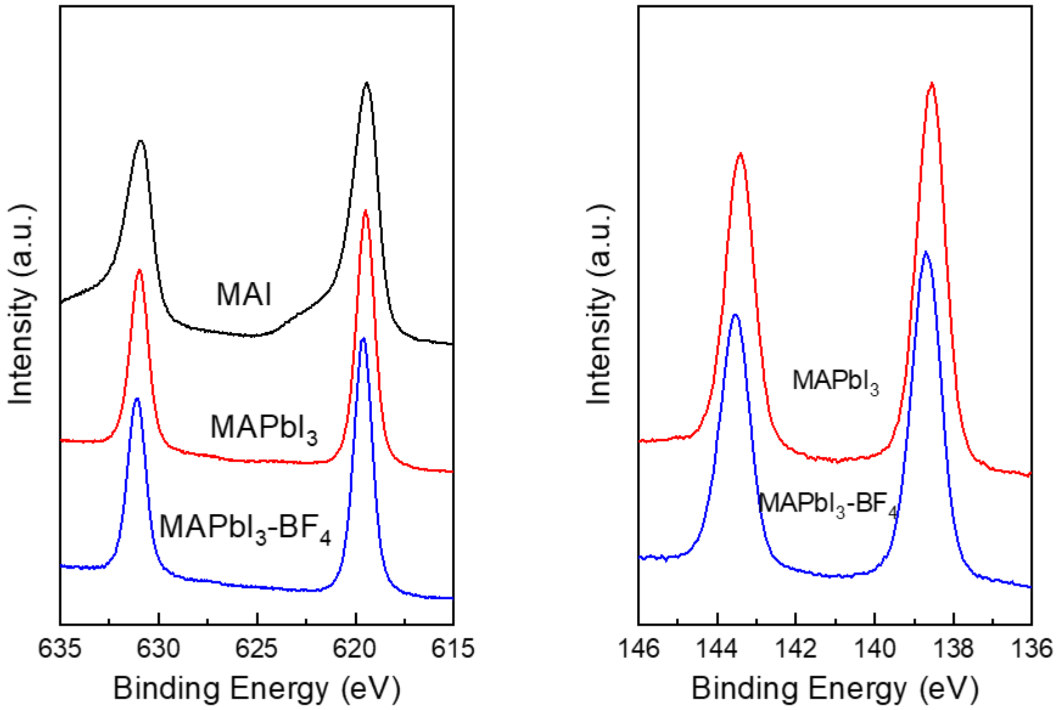

Figure S13. XPS of $MABF_4$ treated and non-treated $MAPbI_3$ perovskite (I 3d and Pb 4f).

References:

[1] A. Al-Ashouri, A. Magomedov, M. Roß, M. Jošt, M. Talaikis, G. Chistiakova, T. Bertram, J. A. Márquez, E. Köhnen, E. Kasparavičius, S. Levcenco, L. Gil-Escrig, C. J. Hages, R. Schlatmann, B. Rech, T. Malinauskas, T. Unold, C. A. Kaufmann, L. Korte, G. Niaura, V. Getautis, S. Albrecht, *Energy Environ. Sci.* **2019**, *12*, 3356.

[2] I. L. Braly, D. W. Dequilettes, L. M. Pazos-Outón, S. Burke, M. E. Ziffer, D. S. Ginger, H. W. Hillhouse, *Nat. Photonics* **2018**, *12*, 355.

[3] I. Schnitzer, E. Yablonovitch, C. Caneau, T. J. Gmitter, *Appl. Phys. Lett.* **1993**, *62*, 131.

[4] L. J. Phillips, A. M. Rashed, R. E. Treharne, J. Kay, P. Yates, I. Z. Mitrovic, A. Weerakkody, S. Hall, K. Durose, *Sol. Energy Mater. Sol. Cells* **2016**, *147*, 327.

[5] J. M. Richter, M. Abdi-Jalebi, A. Sadhanala, M. Tabachnyk, J. P. H. Rivett, L. M. Pazos-Outón, K. C. Gödel, M. Price, F. Deschler, R. H. Friend, *Nat. Commun.* **2016**, *7*, 1.
